# Asymmetrical Update of Beliefs About Future Outcomes is Driven by Outcome Valence and Social Group Membership

**DOI:** 10.5334/irsp.647

**Published:** 2023-04-17

**Authors:** Mihai Dricu, Stephanie Bührer, Dominik A. Moser, Tatjana Aue

**Affiliations:** 1 Biological and Social Emotion Psychology Unit, Institute of Psychology, University of Bern, Bern, Switzerland

**Keywords:** belief update, optimism bias, stereotypes, in-group, out-group, stereotype content model

## Abstract

People are eager to update their beliefs, such as a perceived risk, if they receive information that is better than expected but are reluctant to do so when the evidence is unfavourable. When estimating the likelihood of future outcomes, this phenomenon of asymmetrical belief update helps generate and maintain personal optimism bias. In this study, we investigated whether asymmetrical belief update also extends to estimating the future of other individuals. Specifically, we prompted respondents to assess the perceived likelihood of three social targets experiencing future positive and negative events: An in-group, a mild out-group, and an extreme out-group. We then provided the respondents with feedback about the base rates of those events in the general population and prompted them to re-assess their initial estimates for all social targets. Respondents expected more positive than negative outcomes for the in-group and the mild out-group, but more negative outcomes for the extreme out-group. We also found an asymmetrical update of beliefs contingent on the valence of the future event and the social target. For negative outcomes, respondents updated more following good news than bad news, particularly for the mild out-group. For positive outcomes, respondents equally updated their beliefs following good news and bad news for the in-group and the mild out-group. However, they updated their beliefs significantly more following bad news than good news for the extreme out-group member. Our data thus reveal the strong influence of social stereotypes on future expectancies for others.

## 1 Introduction

Humans generally desire to know what their future holds. Will we be rich, famous, influential, healthy in the future? While we are curious to see what our future holds, we do not treat available background information neutrally. By contrast, we strongly prefer and more easily integrate incoming information that justifies an optimistic rather than a pessimistic outlook. Specifically, we update our beliefs (i.e., initial expectancies regarding our future) more readily when the information we receive is better (good news) rather than worse (bad news) than expected. For instance, people being informed that the likelihood of incurring cancer is higher than they initially believed (i.e., bad news) will have a harder time updating their expectancies than people being informed that the likelihood of incurring cancer is lower than they initially believed (good news). This asymmetric integration of new information into existing belief structures is thought to be almost exclusively driven by the reluctance to update beliefs in face of unfavorable information (Dricu, Kress, et al., 2020), and it helps generate and maintain (over)optimistic beliefs (Sharot & Garrett, 2016; Sharot, Kanai, et al., 2012; Sharot et al., 2011; see also Kress et al., 2018; Singh et al., 2020, for attentional processes likely involved in updating beliefs).

Because people do not only display optimistic biases for themselves but also for individuals and social groups they like or identify with (Aue, Dricu, et al., 2021; Aue et al., 2012; Moser et al., 2020), we investigated, with the current study, whether belief updating asymmetries also arise in the social domain. Specifically, we examined whether the asymmetrical belief update manifested for the self (Garrett & Sharot, 2017; Kuzmanovic et al., 2016; Sharot, Kanai, et al., 2012) extends to social targets (i.e., individuals belonging to different social groups).

Previous studies have used a similar other of ‘the same age, sex and socioeconomic background’ as the respondent to anchor the decision-making process, that is, how one views oneself and one’s future *in comparison* to a similar other (e.g., Kuzmanovic et al., 2015; Kuzmanovic et al., 2019). As such, the main purpose of a similar other was to highlight the personal update of beliefs in an optimistic direction. By contrast, we intended to investigate the ‘other’ as the main unit of analysis. To the best of our knowledge, only a single study has taken a somewhat similar approach. Specifically, Kappes et al. (2018) showed that asymmetrical belief update extends to friends but not to strangers, that is, an unknown individual identified by a picture and a name (Kappes et al., 2018). Notably, though, the study by Kappes et al. did not define the social target beyond the colloquial terms ‘friend’ and ‘stranger’.

In the current study, therefore, we used a formal framework to operationalize the social targets. To this end, we referred to the Stereotype Content Model (SCM), a social psychological model that states that people think and feel about others in terms of two orthogonal dimensions, *perceived warmth* – how (un)likeable someone is – and *perceived competence* – how (un)respectable someone is (Abele & Wojciszke, 2013; Caprariello et al., 2009; Cuddy et al., 2007, 2008; Fiske et al., 2007; Judd et al., 2005; Kervyn et al., 2015; Wojciszke et al., 2009). According to the SCM, in-group members are perceived as high in warmth and competence, whereas out-groups can be placed on a continuum by virtue of their combined warmth and competence (Cikara & Fiske, 2011; Cuddy et al., 2007; Cuddy et al., 2009; Fiske, 2015). For example, there can be ‘mild’ out-groups (warm but not competent; e.g., the elderly and individuals with disabilities [Cuddy et al., 2005; Fiske, 2017]) and ‘extreme’ out-groups (not warm and not competent; e.g., drug and substance abusers [Cuddy et al., 2007]). An extension of the SCM, the Behavior from Intergroup Affect Stereotypes (BIAS) Map (Cuddy et al., 2007, 2008) describes emotional reactions and behavioral intentions associated with different combinations of attributed warmth and competence: Typical social emotions associated with each type of out-group encapsulate pity and sympathy for the warm-incompetent out-group, and contempt and disgust for the cold-incompetent out-group, whereas pride should be experienced with respect to the in-group (see also Caprariello et al., 2009).

Stereotypes and the prejudices that they ensue are a set of motivationally driven and rationally justified emotional reactions and behavioral tendencies (e.g., to help and assist or to impede and marginalize) to maintain the status quo of groups and the society (Cuddy et al., 2007, 2008; Dixon et al., 2012; Fiske, 2015, 2017). For example, individuals who are substance addicts and go on welfare are shamed and ostracized for ostensibly cheating the social contract that so many adhere to (e.g., indignation over not having the will and patience to hold on to a job, not having responsibilities or giving back to society) and for being unreliable and unpredictable to peers (e.g., apprehension that they may steal to obtain their drug). Previously, we showed that such stereotypes play a significant role in optimistic belief *formation* (Dricu et al., 2018; Dricu et al., 2022; Dricu, Schüpbach, et al., 2020). Concretely, we asked participants to rate the chances of different social targets experiencing an identical set of future events. The in-group was chosen to be an extension of oneself (represented by a student character, supposedly warm and competent). A mild out-group (represented by an elderly, supposedly warm but not competent) was chosen to instil compassion and empathic concern, and an extreme out-group member (represented by an alcoholic, supposedly not warm and not competent) was supposed to prompt contempt and denigration. Participants believed that the in-group and the mild out-group member had significantly higher chances of experiencing positive outcomes than negative outcomes, but they believed the opposite for the extreme out-group member. With the current study, we took the logical next step and examined how the same stereotypes influence optimistic belief *update*.

### 1.1 Hypotheses

The SCM/BIAS Map has had a considerable track record in making predictions about out-group members (e.g., Cuddy et al., 2009; Dricu et al., 2018; Dricu, Schüpbach, et al., 2020; Fiske et al., 2007). Previously, we showed that respondents evaluate the in-group member qualitatively differently from out-group members, above and beyond warmth and competence traits (Dricu et al., 2020). Respondents evaluate in-group members positively compared to the out-group members because of their similarity to oneself (Dricu, Schüpbach, et al., 2020; Moran et al., 2006; Morrison et al., 2012) rather than their traits of high warmth and high competence (see Harris & Fiske, 2006, 2007, for such argumentation). Moreover, it appears that, as far as predictions for the out-groups are concerned, the combinations of the warmth and competence dimensions give rise to three unique quadrants whose properties are more than the expected cumulative effects of their underlying SCM dimensions, as behavioral data and neuroimaging findings indicate (Dricu et al., 2020). Put differently, the particular combination of warmth and competence traits becomes a unique set of attributes that are not shared with another quadrant that have either the warmth or the competence dimension in common. This can also explain the qualitatively different emotional responses observed for social targets belonging to the different quadrants. Specifically, the combination of high warmth and low competence (e.g., elderly people) generates a particular attitude of compassion and empathic concern toward this type of out-group (e.g., Cuddy et al., 2007; Cuddy et al., 2005; Dricu, Schüpbach, et al., 2020). By contrast, the combination of low warmth and low competence for extreme out-group members leads to contempt and denigration. Alcoholic characters and other members belonging to the low-competence and low-warmth quadrant are seen as doubly unworthy of societal (i.e., common) resources: due to their general lack of likeability and trustworthiness (i.e., low warmth) and on account of their inability to contribute back to society for the received goods (i.e. low competence; cf. Cikara et al., 2010; Cuddy et al., 2007).

We assumed these different emotional responses to trigger selective updating of beliefs toward the three SCM characters. Specifically, we expected a stronger update for the in-group member and the mild out-group member following good news compared to bad news (H1a; see Table 1). In light of recent neuroimaging evidence (Dricu, Schüpbach, et al., 2020), however, the underlying cognitive mechanisms are quite different for the in-group (i.e., self-referential processing) and the mild out-group (i.e., empathic concern), despite the shared behavioral pattern for belief formation. Because the mild out-group uniquely induces compassion and empathic concern (Dricu, Schüpbach, et al., 2020), it may be the social target that triggers the strongest helping behavior in an observer. In the current study, active helping behavior was prohibited; helping behavior therefore may have taken a more indirect form, reflected in wishing those targets a particularly good future. We hence expected that the difference in update between good news and bad news would be even higher for the mild out-group compared to the in-group (H1b). Regarding the extreme out-group, we predicted that belief update would be stronger following bad news compared to good news (H2). This is because the low competence-low warmth combination of traits uniquely generates feelings of contempt and elicits demeaning and neglectful attitudes or active rejection (Caprariello et al., 2009; Cuddy et al., 2007). As such, individuals are emotionally invested in imagining the worst about these social targets. Finally, we expected that these patterns would hold true for both positive and negative events (H3), in line with previous claims for the personal domain (Garrett & Sharot, 2017).

**Table 1:** Summary of the hypotheses.


CHARACTER	POSITIVE EVENTS	NEGATIVE EVENTS

In-group	Update good news > Update bad news	Update good news > Update bad news

Mild out-group	Update good news >> Update bad news	Update good news >> Update bad news

Extreme out-group	Update good news < Update bad news	Update good news < Update bad news


*Note*: >> refers to a larger update following good news than bad news compared to >.

## 2 Materials and Methods

### 2.1 Participants

One hundred and thirty-three Swiss university psychology students participated in this study (age: *M* = 21.9 years, *SD* = 2.24 years; 107 female, 26 male). They were recruited via the local participant pool at the University of Bern and received course credits for their participation. As participation inclusion criteria, respondents had to be German-speaking full-time university students aged between 18 and 35 years. We used the GPower 3.1 software (Erdfelder et al., 1996) to calculate the desired sample size. Because the only other two studies using the same linear mixed models (LMMs) as us (Garrett & Sharot, 2017; Marks & Baines, 2017) do not provide effect sizes, we based our power calculation on a repeated-measures design, which many previous studies on belief update have used (e.g., Garrett & Sharot, 2014; Garrett et al., 2014; Moutsiana et al., 2015; Moutsiana et al., 2013). Furthermore, because our design does not measure belief update for oneself but for social groups, we looked at the only other study investigating belief updates toward a social target (Kappes et al., 2018, Study 1, friend as a social target). Kappes et al. found a small effect size of 0.29, and with a power of 0.90 we needed a sample of at least 90 participants. LMMs are more powerful than analyses of variance (ANOVAs; e.g., Baayen et al., 2008; McGann & Speelman, 2013; Wang & LA Goonewardene, 2004), wherefore a power analysis for LMM based on ANOVA specifications yields a conservative estimate. Nevertheless, our sample size of 133 is higher than the minimum suggested by GPower. Furthermore, because we treat both respondents and scenarios as random effects (see section 3.2.1 for details), we are confident that our study is well-powered and its findings can be generalized beyond our sample of respondents and scenarios. All participants gave informed and written consent for their participation. The local ethics committee of the University of Bern approved all experimental protocols and methods of data collection, data handling and data analysis. Furthermore, all methods and experimental protocols were performed fully in line with the Declaration of Helsinki (Carlson et al., 2004).

### 2.2 Procedure

The task was adapted from Dricu et al. (2018). Participants were told that the aim of the current study consisted of testing newly developed stimulus material for its use in subsequent studies without any mention of beliefs, stereotypes, or perceptions of warmth and competence. In short, participants evaluated the likelihood that three different target characters would face each of twenty-four identical events. The characters had been validated as an implicit in-group (a student of psychology, high warmth/high competence) and two out-groups: a mild out-group (an elder, high warmth/low competence; the elder character was hence more or less similar to the student character in terms of perceived warmth but different in terms of perceived competence) and an extreme out-group (an alcoholic, low warmth/low competence; this character differed from the student character both in terms of warmth and competence). We did not offer our participants any further information about the characters under investigation because we hypothesized that implicit group evaluations would suffice to evoke the assumed stereotypes of warmth and competence as well as the associated behavioral and emotional responses revealed in earlier findings (e.g., Cuddy et al., 2007, 2008; Dricu et al., 2018; Dricu, Schüpbach, et al., 2020).^1^

Twelve desirable and twelve undesirable events (details see section 3.1 and Table A1 in the appendix) were used out of the original set of thirty-two events (Dricu et al., 2018) to keep the experiment length to a minimum. The average perceived frequency in the general population expressed by participants of that earlier study was used as feedback information (so-called base rates) in the current study. To avoid possible gender influences, a male and a female version were created. Female participants saw female animated characters while male participants viewed male animated characters. The resulting 144 character × event combinations (3 characters × 2 genders × 24 events) were created using The Sims 4 (Electronic Arts, California, USA).

**Figure 1:**
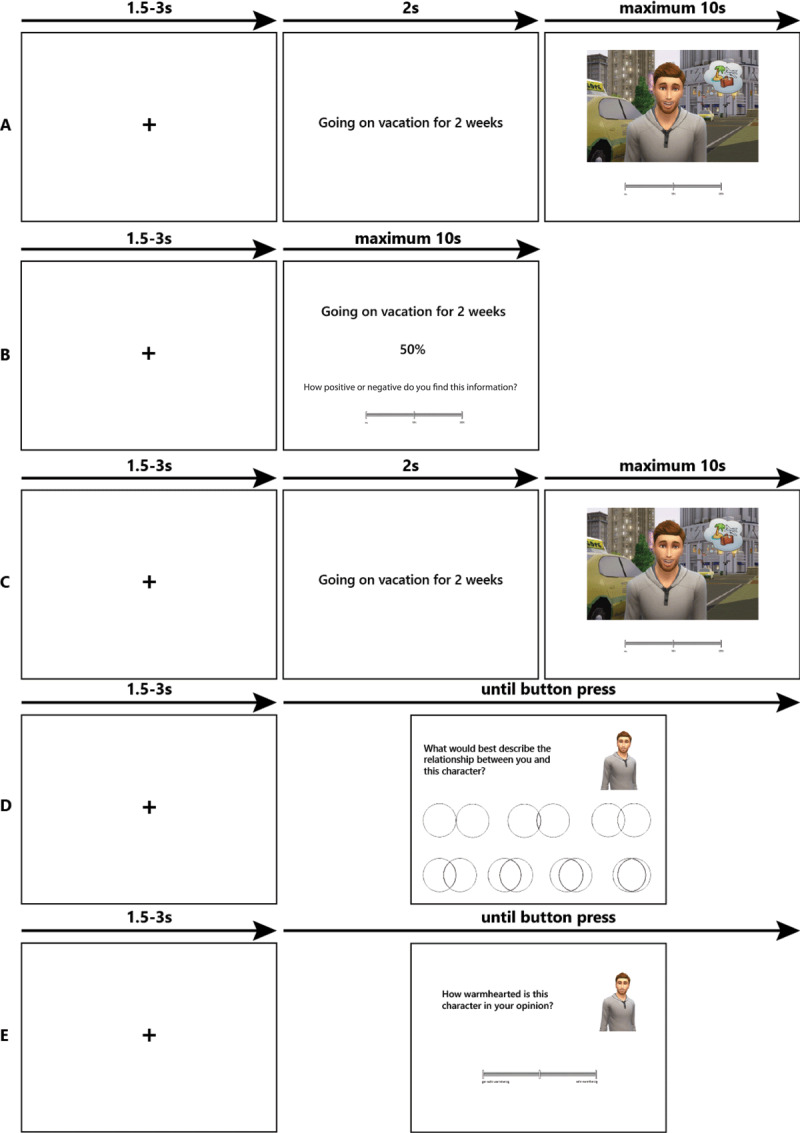
Depiction of the experimental paradigm. *Note*: In the first part, respondents estimated the likelihood of each of the three characters experiencing each of the 24 target events in a fully randomized manner **(A).** Respondents saw the base rates for each target event in the general population and were asked to assess how positive or negative they found this information (feedback task); **(B).** Respondents were asked to re-estimate the likelihood of each character experiencing each event **(C).** Finally, respondents expressed the degree of identification with each character **(D)** and the perceived warmth and competence **(E).**

The experiment was delivered to the participants with E-Prime 2.0 Professional (version 2.0.10.353; Psychology Software Tools, Pittsburgh, USA) and consisted of four parts (Figure 1), each preceded by written instructions displayed on the computer screen. In the first part, respondents estimated the likelihood of each of the three characters facing each of the twenty-four events in a fully randomized manner. They used a visual analogue scale from 0% (will certainly not happen) to 100% (will certainly happen). In the second part, feedback was provided: The respondents were informed that they would see each of the twenty-four events again, paired with the likelihood estimate for the general population (obtained from a large survey). They were instructed to think about the association between each event and the percentage shown and give their opinion as to how positive or negative they found the information (e.g., how positive or negative they found the likelihood of being bitten by a dog for the general population). The purpose of the inclusion of these questions was to have the respondents engage with base rates to ensure a minimum level of incidental encoding of the information. Importantly, feedback was given for the general population and not separately for each character. In the third part, the respondents were asked to rate again the likelihood of each of the three characters in each of the twenty-four events, also in a fully randomized manner. The fourth and last part of the e-Prime experiment consisted of ratings of warmth and competence of each of the three characters, along with ratings of social identification with the different characters as assessed with the Inclusion of the Other in the Self Scale (Aron et al., 1992). Both served as manipulation checks (Supplementary Analysis S1). The average length of the e-Prime experiment was 18 minutes, and it was preceded (~5 minutes) and (mostly) succeeded by online questionnaires assessing personality traits and dispositional optimism (total study duration: ~65 minutes). The data from these questionnaires were unrelated to the present aims and therefore are not presented here.

## 3 Analysis and Results

### 3.1 Selection of Stimuli

The twelve positive and twelve negative events were selected from the larger sample of thirty-two events from (Dricu et al., 2018). We incorporated both positive events and negative events that were matched on several characteristics shown to also influence the size of an optimistic bias displayed. Among those potential confounders are: a) event controllability (degree to which the positive and negative consequences of an event can be influenced by personal or communal actions [Klein & Helweg-Larsen, 2002]), b) emotional arousal (relating to the intensity of the emotional responses evoked by the event [Krizan & Windschitl, 2007; Lench, 2009]), c) personal/prior experience (earlier exposure to the event [Cho et al., 2010; DeJoy, 1989; Helweg-Larsen & Shepperd, 2001]), and d) event frequency (i.e., how often the event occurs in the general population [Chambers et al., 2003; Klar & Ayal, 2004; Price et al., 2002]). Any of these characteristics alone can introduce potential confounds we wanted to rule out. Our study hence matched positive and negative events on strength of valence (i.e., average deviation of the positive and negative scenarios from the neutral midpoint), perceived frequency, controllability, emotional arousal and personal experience with the events.

The selected positive and negative events (Table A1) did not differ with respect to those potential confounders (all analyses were conducted with Jamovi [The jamovi project (2020). jamovi. (Version 1.2) [Computer Software]. Retrieved from https://www.jamovi.org.]). Specifically, they did not differ in terms of *perceived controllability* (*t* (11) = –0.076, *p* = 0.941), *emotional arousal* (*t* (11) = 0.991, *p* = 0.343), or *personal experience* (t (11) = 1.501, *p* = 0.162). Furthermore, as intended, the positive and negative events differed in terms of their *emotional valence* (*t* (11) = 11.557, *p* < 0.0005), but were matched on the *distance from the hypothetical “neutral” point* (*t* (11) = –0.81, *p* = 0.941). Most importantly, the *feedback values* (i.e., perceived frequencies of the events in the general population; so-called base rates) presented to the participants did not differ between positive and negative events (*t* (11) = .381, *p* = 0.711).

The distribution of the feedback (base rates) was normal for negative events (Shapiro-Wilk *W* = 0.961, *p* = 0.801, *M* = 60.1%, *SD* = 7.97%) but binomial for positive events (Shapiro-Wilk *W* = 0.805, *p* = 0.011, *M* = 61.4%, SD = 11.3%; Supplementary Figure S1). However, LMMs are very robust to violations of normality (Schielzeth et al., 2020), and including the estimation errors (i.e., the difference between the feedback presented and the participant’s initial likelihood estimate [before receiving the feedback information]) into the analysis as covariates would bypass an artificial bias, as described by (Garrett & Sharot, 2017).

### 3.2 Belief Updating Results

#### 3.2.1 Model specifications

We performed an LMM (Supplementary Table S1) with the update scores as the dependent variable and the following predictors: character (in-group, mild out-group, extreme out-group), valence of scenario (positive, negative), valence of feedback (good news, bad news) and their interactions. Additionally, we considered the following covariates: estimation error (main effect), the first estimate (main effect), and the interaction terms between the character and estimation error and between the character and the initial estimate.^2^

Specifically, we used LMM as implemented in the GAMLj module in Jamovi (https://www.jamovi.org.). The reason for our preference of this statistic over traditional methods is that it permits to account for both participant- and scenario-related variance in the same model (see Baayen et al., 2008, for a more detailed description of the rationale for using this approach, and Grindrod & Raizen, 2019, for a recent implementation of LMM to repeated-measures designs).

Our LMM had a completely crossed design, with crossed random effects for participants (n = 133) and scenarios (n = 24), which were both at level 2, and with the update scores for each of the three target characters within each combination of participant and scenario (level 1 outcome data). Target character (in-group, mild out-group, extreme out-group) and valence of feedback (good news, bad news) were level 1 categorical predictors, and valence of scenario (positive, negative) was a level 2 categorical predictor, as it represented a characteristic of the scenarios only. Estimation error and initial estimate were level 1 continuous predictors (covariates centered on the mean).

We note that, for the purpose of the analysis, the trials were classified as good news or bad news at the participant level, depending on the type of feedback received (i.e., base rate presented). For positive scenarios, trials were labelled good news/bad news if the base rate was higher/lower than what the respondent had initially estimated, regardless of the character. By contrast, for negative events, trials were labelled good news/bad news if the base rate was lower/higher than what the respondent had initially estimated, regardless of the character. Calculation of the update values followed these classifications (Supplementary Table S2) such that stronger updating in the direction of the feedback resulted in more positive update values. Conversely, negative update values resulted when a participant’s second estimate even more strongly deviated from the feedback presented than the first estimate.

#### 3.2.2 Findings

We assumed different emotional responses to the target characters (resulting from particular combinations of perceived warmth and competence) to trigger selective updating of beliefs. Concretely, we expected a stronger update for the in-group member and the mild out-group member following good news compared to bad news (H1a; Table 1), with the effect being more pronounced for the mild out-group (H1b). By contrast, for the extreme out-group member, we predicted that belief update would be stronger following bad news compared with good news (H2). Finally, we expected that these patterns would hold true for both positive and negative events (H3).

**Figure 2:**
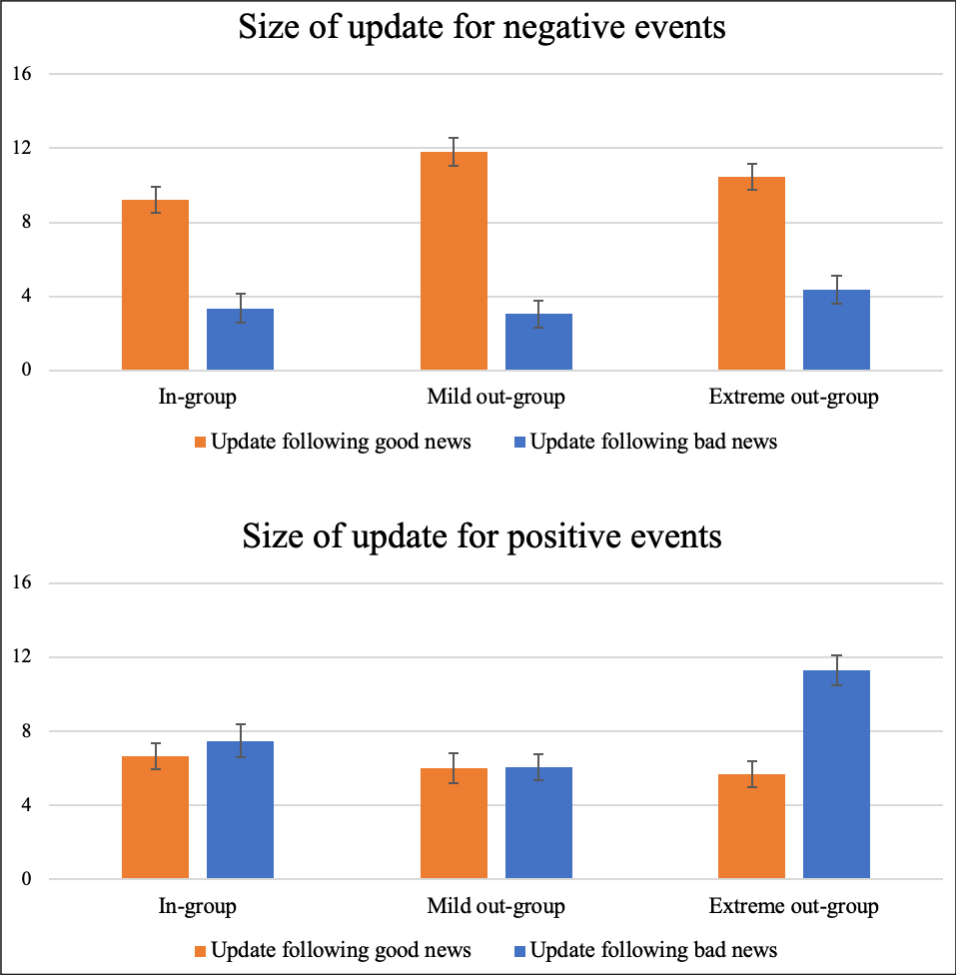
Size of update for negative and positive events. *Note*: The y axis represents the magnitude of the update: the difference in likelihood between the initial estimate (in %) and the second estimate (in %).

There was a main effect of character, *F*(2,9441.77) = 7.39, *p* < 0.001, qualified by a three-way interaction with valence of scenario and valence of feedback, *F*(2, 9452.11) = 3.84, *p* = 0.021 (Figure 2, Tables 2 and 3). This significant interaction revealed that effects for positive and negative scenarios were not comparable; as such, this finding thus ran counter our H3 (similar pattern of response for positive and negative events). We resolved the interaction first by valence of scenario and then by character. For negative events, respondents generally updated their beliefs significantly more following good news (*M* = 10.49%) than bad news (*M* = 3.58%) for all characters, *F*(2, 1680.35) = 96.94, *p* < 0.001, which is consistent with H1a, but not with H2 (for the extreme out-group stronger updating had been expected in response to bad compared with good news). Furthermore, consistent with our H1b, our participants updated the most for the mild out-group (*M*_diff_ = 8.75%), followed by the extreme out-group (*M*_diff_ = 6.1%) and the in-group (*M*_diff_ = 5.86%; Figure 2, Tables 2 and Table 3).

**Table 2:** Descriptive statistics for each of the twelve experimental conditions.


CHARACTER	SCENARIO	FEEDBACK	FIRST ESTIMATE *M* (*SD*)	UPDATE *M* (*SD*)	ESTIMATION ERROR *M* (*SD*)

Extreme out-group	Negative	Bad news	37.7 (18.0)	4.23 (16.7)	22.8 (16.1)

Extreme out-group	Negative	Good news	79.7 (12.7)	10.1 (15.0)	19.9 (12.7)

Extreme out-group	Positive	Bad news	77.0 (15.7)	9.36 (16.3)	16.6 (11.6)

Extreme out-group	Positive	Good news	33.2 (18.8)	7.43 (16.5)	28.1 (16.5)

Mild out-group	Negative	Bad news	31.3 (17.2)	4.46 (15.9)	29.4 (17.3)

Mild out-group	Negative	Good news	75.4 (13.8)	10.6 (16.1)	16.4 (11.5)

Mild out-group	Positive	Bad news	83.3 (13.5)	5.71 (12.6)	19.5 (11.0)

Mild out-group	Positive	Good news	35.5 (20.3)	5.29 (14.8)	20.8 (14.5)

In-group	Negative	Bad news	35.5 (18.6)	4.14 (15.6)	25.2 (17.5)

In-group	Negative	Good news	79.0 (14.4)	8.54 (14.2)	19.4 (12.0)

In-group	Positive	Bad news	83.0 (13.9)	6.18 (12.7)	21.1 (12.0)

In-group	Positive	Good news	40.6 (20.9)	6.16 (15.4)	17.1 (14.1)


*Note*: The values represent the mean ratings based on the sample of 133 respondents.

**Table 3:** Linear Mixed Model: Fixed effects Omnibus tests.


PREDICTOR	*F*	df	DEN df	*p*

Character	7.39	2	9441.8	<.001

Valence scenario	0.09	1	23.3	0.764

Valence feedback	69.03	1	8434.3	<.001

Estimation error	763.74	1	9288.7	<.001

Initial estimate	1.08	1	1027.1	0.301

Character × Valence scenario	6.56	2	9387.0	0.001

Character × Valence feedback	15.65	2	8807.5	<.001

Valence scenario × Valence feedback	66.05	1	3678.5	<.001

Character × Estimation error	0.95	2	9098.3	0.385

Character × Initial Estimate	0.76	2	9353.4	0.470

Character × Valence scenario × Valence feedback	3.84	2	9452.1	0.021


For positive events, participants equally updated in response to good news and bad news for the in-group, *t*(9169) = 0.76, *p* = 0.447, and the mild out-group, *t*(9345) = –0.05, *p* = 0.959. However, they updated significantly more following bad news than good news for the extreme out-group, *t*(6517) = –5.44, *p* < 0.001. In other words, for positive situations, participants displayed an asymmetrical update of beliefs only for the extreme out-group. This finding supports hypothesis H2 concerning the extreme out-group member for positive events, but not hypothesis H1a, because we expected a higher update following good news than bad news for the in-group and the mild out-group member (Table 1).

In sum, therefore, findings for the negative events support hypotheses H1a and H1b concerning the asymmetrical belief update toward the in-group and the mild out-group. However, it does not support hypothesis H2 concerning the extreme out-group. By contrast, for positive events, it was the reverse: H1a and H1b were not supported by the data, but H2 was.

To determine whether the null effects for the in-group and the mild out-group in the positive situations were credible and not simply a result of an underpowered study (i.e., too small sample size), we performed a series of post-hoc Monte Carlo simulations (See Supplementary Analysis S2 and Supplementary Tables S3 and S4). Effect sizes as small as 0.20 standardized mean difference between update to good news vs. bad news for the in-group and the mild out-group were significant in 88% of the simulations at *p* < 0.05, even given the extreme outgroup’s tendency in the opposite direction. Furthermore, the two-way interaction character × feedback for positive events became significant with this setup in 95% of the simulations when the main effect (i.e., the difference between good news and bad news) amounted to 0.15 *SD*s. To be significant, such effects therefore do not need to be large and the present sample size is adequately powered to find significant effects concerning positive events, should they arise. Significant effects that are not directly related to our hypotheses are reported in the Supplementary Material (section 6).

## 4 Discussion

In this study, we investigated whether the phenomenon of asymmetrical belief update about one’s own future extends to estimating the future of other individuals who are more or less close to us. We prompted respondents to assess the likelihood of future positive and negative events of three social targets: an in-group character supposedly triggering pride, a mild out-group character instilling compassion and empathic concern, and an extreme out-group character instilling contempt and denigration. We then provided the respondents with feedback about the base rates of those events in the general population. Lastly, respondents were prompted to re-assess their initial estimates for all social targets.

We hypothesized that respondents would update their beliefs significantly more following good news than bad news for the in-group and the mild out-group members (H1a), and that this pattern would hold for both positive and negative events (H3). The in-group member was designed to be an extension of oneself (i.e., a psychology student character for psychology student respondents) and thus bear a direct relevance and resemblance to oneself (Cuddy et al., 2007; Dricu et al., 2018; Dricu, Schüpbach, et al., 2020; Harris et al., 2000). As such, respondents should update more following good news than bad news, in line with studies on belief update in the personal domain (Dricu, Kress, et al., 2020). The mild out-group was designed to elicit compassion and empathic concern due to their unique combination of high warmth and low competence traits (Cuddy et al., 2007; Cuddy et al., 2005; Fiske, 2017). We predicted a same-directed asymmetrical belief update as for the in-group: Beliefs would be updated more strongly following good news than bad news. Furthermore, because the mild out-group uniquely induces compassion and empathic concern (Dricu, Schüpbach, et al., 2020), we also expected that the difference in update between good news and bad news would be even higher for the elderly target compared to the in-group target (H1b). Our findings support these (H1a and H1b) predictions for negative events but not for positive events (in which respondents equally updated their beliefs following good news and bad news for the in-group and the mild out-group). In addition, the fact that we observed a different pattern of response for positive and negative events is not supportive of our H3.

To our knowledge, no studies have investigated belief update concerning social targets for both positive and negative events, and therefore no direct precedent exists. Three studies have simultaneously looked at positive and negative events in the personal domain and reported the belief update separately (Garrett & Sharot, 2017; Marks & Baines, 2017; Shah et al., 2016). Our findings converge with these studies for negative but not for positive events. When Shah et al. matched the positive and negative events on mean base rates for some experiments (3A and 3B) they found that respondents updated more to good news than bad news for negative events, but the reverse pattern arose for positive events: greater updates to bad news than good news. However, because both the positive and the negative events were overall decidedly rare (positive *M* = 22.10% and negative *M* = 23.38%), one cannot exclude that event rarity may have been behind this pattern of findings. Garrett and Sharot (2017) reported that respondents similarly updated to good versus bad news for positive and negative events, albeit with a much smaller effect size for the former.

These findings combined with our own hence suggest that different cognitive mechanisms may underly the belief update observed for positive vs. negative scenarios. It is possible that the robust optimistically biased update of beliefs for negative events (displayed across studies for the self, the in-group, and the mild out-group) reflects an evolutionary mechanism driven by the human need to reduce the anxiety associated with the risk of facing undesirable negative outcomes (Krizan & Windschitl, 2007, 2009; Lench, 2009). As such, one may be highly motivated to revise estimates following good news (i.e., the risk of a negative outcome is lower than anticipated), but reluctant to revise following bad news (i.e., the risk is higher than anticipated). By contrast, for positive events such a hard-wired mechanism may not exist (possibly because positive outcomes are not life- or relationship-endangering), wherefore individuals equally update to different types of new information (but note the divergent findings of Shah et al., 2016, and Garrett & Sharot, 2017).

We note that, in our study, the amount of belief update toward the in-group and the mild out-group was similarly low for positive events following good news and bad news, and for negative events following bad news. This suggests that, instead of a selective reluctance to update to bad news, above and beyond event valence (e.g., Sharot & Garrett, 2016), people are in general wary of altering prior beliefs, with the exception of encountering good news for negative events.

We further hypothesized that respondents would update significantly more following bad news than good news for the extreme out-group member (H2). This prediction was only supported when looking at the positive events. For negative events, respondents updated more to good news than to bad news. SCM and its BIAS Map extension posit that endangered access and utilization of (common) resources is the driving force behind management of impressions about others, informing the perceived warmth traits (Cikara et al., 2010; Cikara & Fiske, 2011; Cuddy et al., 2007). Alcoholic characters and members belonging to the low-competence and low-warmth quadrant are seen as mostly exploiting these resources. As such, one can think of the positive outcomes/events in our study as a proxy for the future access and utilization of (common) resources. If the alcoholics are seen as undeserving of these resources (positive outcomes; Cuddy et al., 2007, 2008), then respondents are motivated to think about them as not being able to access them. Consequently, respondents update more following bad news (i.e., alcoholics are less likely to access positive resources) than good news (i.e., alcoholics are more likely to access positive resources).

The fact that respondents updated more following good news than bad news for the extreme out-group in negative situations was unexpected. Because our participants did not reveal updating in response to bad news for both the in-group and the mild out-group either, one possibility is that respondents are reluctant to update in general to bad news and, thus, the social target is unimportant. However, the pattern observed for the extreme out-group regarding positive events does not support such an interpretation. A more likely possibility, therefore, is that respondents are motivated to think about the extreme out-group members as having restricted access to positive resources (Cuddy et al., 2007, 2008), but they are not motivated enough to imagine them in worse-than-already-imagined predicaments. Consistent with this pattern, individuals reveal reduced empathy with alcoholics for positive but not for negative events (Aue, Bührer, et al., 2021; for similar observations regarding cocaine use, see Aue et al., 2022). We note that participants already expected more negative than positive outcomes to happen to these individuals (Dricu et al., 2018; see also Supplementary Analysis S3). Updating toward increased likelihood of negative outcomes would perhaps amount to unreasonable cruelty. However, such a reflection does not readily explain why the inverted pattern is observed, (i.e., greater update following good news than bad news for the extreme out-group regarding negative events). We speculate that respondents may become aware of their uniquely harsh expectations toward the extreme out-group members upon the received feedback and may be eager to correct them. The analysis of the desirability bias for the extreme out-group before and after the feedback supports such an interpretation: Respondents in the current study were less biased following feedback (Supplementary Analysis S4), but the desirability bias prevailed, nevertheless.

### 4.1 Strengths and Limitations

Our study serves as a proof of concept for an alternative method of delivering the feedback in the belief update paradigm. In virtually all studies on belief update, a feedback is delivered immediately after each initial estimate and the participant is subsequently prompted to adjust their estimate immediately after the feedback (e.g., Kuzmanovic et al., 2015; Kuzmanovic & Rigoux, 2017; Kuzmanovic et al., 2018) or in a later session (e.g., Garrett et al., 2018; Garrett & Sharot, 2014; Kappes et al., 2018; Moutsiana et al., 2015; Moutsiana et al., 2013; Sharot, Guitart-Masip, et al., 2012; Sharot, Kanai, et al., 2012; Sharot et al., 2011). Such elegant design is possible when there is only one target to evaluate, oneself (although see Garrett & Sharot, 2017; Garrett et al., 2014; Kappes et al., 2018), or two alternating targets, oneself and a stranger (Kappes et al., 2018), and oneself and the average person (Garrett & Sharot, 2017; Garrett et al., 2014). However, we wanted to investigate how respondents update their beliefs in response to three targets: an in-group member (high on warmth and high on competence), a mild out-group member (high on warmth and low on competence) and an extreme out-group member (low on warmth and low on competence). Adhering to the original paradigm could have been tedious for the participant, as updating three consecutive estimates immediately after the feedback could induce frustration or boredom, potentially masking the effects of the paradigm. In the present study, therefore, participants made their first estimate unhindered for all three characters in all scenarios (in a randomized order), and then engaged actively with the feedback information. In the last part, participants revised their initial estimates for all three characters again in a randomized order. Yet, future investigations should examine whether giving direct or delayed feedback produce different updating effects.

The current study is the first to investigate the updating phenomenon using a set of positive and negative events that had been matched on four crucial characteristics, that is, perceived controllability and frequency in the general population, emotional arousal, and personal experience. This matching reduces the potentially confounding effects that any of these characteristics can have on belief update and optimism bias (e.g., Dricu, Kress, et al., 2020; Helweg-Larsen & Shepperd, 2001; Klein & Helweg-Larsen, 2002), and provides a better assessment of the extent of the asymmetrical belief update. While the positive and negative events were matched on these crucial characteristics, this came at the expense of a reduced pool of events: twelve positive and twelve negative. To strengthen the conclusions drawn from our experiment, we encourage future studies to use a higher number of events. These studies could also test the phenomenon of belief update for neutral (or less emotional) situations, that are also matched in key characteristics with the positive and negative events. Such a design could more definitively test the extent of the belief update phenomenon.

We note that we did not formally test for memory errors, namely differences in recalling the presented feedback percentages for negative and positive events. It is possible that the valence of the event (or an interaction of the valence of the event with the valence of the feedback) may induce different rates of remembering the feedback values, thus influencing the subsequent update. For example, one may better remember the feedback value during negative events following good news than during positive events following bad news. However, to the best of our knowledge, none of the studies on belief update collecting information on memory errors have found statistically significant differences in memory errors between positive and negative events (Garrett & Sharot, 2014; Garrett et al., 2014; Moutsiana et al., 2015; Sharot, Guitart-Masip, et al., 2012; Sharot, Kanai, et al., 2012; Sharot et al., 2011). As such, we are confident that our findings were not affected by systematic differences in memory errors between positive and negative events.

Moreover, we interpret many of the findings through the prism of theoretically assumed stereotypes and prejudices, including behavioral tendencies and emotions. For example, previous neuroimaging and behavioral studies have shown that individuals perceived as warm but not competent are associated with pity (Cuddy et al., 2007), compassion and empathy (Aue, Bührer, et al., 2021; Dricu et al., 2018). Members of social groups perceived as cold and not competent are associated with contempt and disgust (Cuddy et al., 2007; Dricu et al., 2018). Because we did not directly collect data on the emotions and behavioral tendencies hypothesized by the SCM/BIAS Map, we acknowledge this as a limitation of our study. Future studies should confirm our theoretically assumed interpretations.

We also note that our study investigated updating in a very specific population, namely psychology students. However, not all students share the same values and attitudes, which may impact the power of stereotypes as well as extent and type of social prejudice displayed (Dambrun et al., 2009; Guimond & Palmer, 1996). Furthermore, two factors may prevent the generalization of our results: First, the study sample was a convenience sample, with women being over-represented. Second, there is a certain risk, that the effects reported in the current study are target-dependent (i.e., restricted to student, elderly, and alcoholic targets). Future investigations should hence test whether the observed effects generalize to other types of social targets.

In this study, we used the original paradigm by Sharot and colleagues (2011) to determine whether the feedback received is good or bad. Specifically, good or bad news were defined as the difference between the base rate provided by the experimenter and the first estimate provided by the respondent. We note that this is not the only possible definition and there have been recent attempts to fine-tune the paradigm to correct some of the potential confounds in the original design (Kuzmanovic & Rigoux, 2017; Shah et al., 2016). However, both the recent and the original paradigm were empirically tested and the differences between them were found to be inconsequential (Garrett & Sharot, 2014; Kuzmanovic et al., 2015). More importantly, the recent and the original paradigms converge in their findings: Respondents update their beliefs more strongly when faced with good news than bad news regarding their personal future (Garrett & Sharot, 2014, 2017; Kuzmanovic et al., 2015; Kuzmanovic & Rigoux, 2017; Kuzmanovic et al., 2018; Kuzmanovic et al., 2019). In addition, using the original design over the recent one offers us and other researchers the chance to compare findings across multiple studies. This is particularly important because we already bring significant alterations to the update paradigm. For example, instead of using the self as the unit of analysis, we introduce three social targets; instead of providing immediate feedback (i.e., the base rate) to the respondent, we introduce a delay. Altering the core belief update methodology would have made it more difficult to interpret the results within the larger field of asymmetric belief update. Nevertheless, future studies could replicate our current findings by defining the good news/bad news as the distance between the respondent’s perceived base rate in the general population and the experimentally presented base rate (Kuzmanovic & Rigoux, 2017).

Finally, we might also be criticized on the ground that the respondents evaluated the likelihood estimates of the characters differently already before they received the feedback information (Supplementary Analysis S3). We counter such criticism by pointing to the lack of interaction between the character and the first estimate as well as between character and estimation error in our analysis of the size of updates (Table 3). These findings suggest that putative differences in how our participants evaluated the characters before reception of the feedback did not impact how they updated these initial estimates. However, it still is possible that those initial estimates somehow influenced the relevance of the feedback provided, thereby determining how strongly specific diagnostic information (i.e., stereotypes) potentially overrode the consideration of base rates. The partial disregard of base rates at the expense of perceived diagnostic information is a phenomenon long recognized in psychology (e.g., Christensen-Szalanski & Beach, 1982; Kahneman & Tversky, 1972; Prike et al., 2020) and does not only affect our study. Future investigations should hence examine whether such influences on our reported asymmetry effects exist.

### 4.2 Implications

Humans generally desire to know what their future holds, such as what the side effects of a medication are (Dickinson & Raynor, 2003). Knowledge about future happenings helps us to prepare adaptive actions, thereby maximizing rewards and minimizing punishments. Decidedly, whether we seek out new information and whether we respond appropriately to it have important societal implications in domains ranging from health care to human interactions. Findings on asymmetric belief update suggest that people at times decide to remain ignorant, namely whenever the anticipation of future outcomes is not as rosy as initially desired (positive events, bad news) or even poorer than initially feared (negative events, bad news). For personally relevant scenarios, such a decision may, for instance, involve postponing medical check-ups despite discernible symptoms (Taber et al., 2015), because of a reluctance to face the possibility of severe sickness.

In the social domain, asymmetric updating is even more complex, because its underlying mechanisms rely on the degree to which we like or identify with other people. For example, we may not sufficiently strongly encourage our close ones to undertake the medical check-up, because we shield our attentional system from this negative information or refuse to admit that these symptoms might be indicative of some sort of severe sickness. By contrast, we may overweigh the same symptoms for extreme out-groups such as alcoholics, possibly leading to the premature conclusion that these social targets are deemed anyway. As such, a medical check-up may be considered too late and not capable of helping them.

In fact, our data demonstrate that stereotypes associated with the kind of social target investigated are strong determinants of the kind of asymmetric response observed. These findings in combination with earlier research output (Aue, Bührer, et al., 2021; Dricu et al., 2018) reveal strong discrimination effects toward vulnerable social groups such as substance abusers: We already expect less good/worse outcomes for these targets than for in-group targets or out-group targets we like, we display less empathy toward them, and we are resistant to update our negative view of them. Moreover, unfavourable discrimination as revealed in social beliefs, their updating, and associated empathic responding may feed back to these stigmatized groups and trigger unfortunate self-discrimination processes that induce mental and physical problems (Aue et al., 2022; Matthews et al., 2017; Young et al., 2005). It hence will be very interesting to test whether substance abusers compared with controls show an inversed pattern of belief update asymmetry for their in-group.

Our data point to the possibility that people actively search for and integrate information in their belief structures that justifies their attitudes toward the different social groups under investigation. Such asymmetric updating reinforces the view of social groups as distinct entities and will render it especially difficult for stigmatized out-group members to conquer social borders, evoke positive attitudes, or join another (more positively evaluated) social group.

The motivation to stick with positive views of the in-group and negative views of disliked out-groups may be founded in the human need for a positive social identify (Abrams & Hogg, 1990; Tajfel et al., 1971; Turner & Onorato, 2014). Overestimating the likelihood of positive outcomes/underestimating the likelihood of negative outcomes for in-group peers may function (via identification processes) as an affective reward and serve to raise the experienced positivity of one’s own identity (Aue, Dricu, et al., 2021; Aue et al., 2012). Importantly, positive distinction processes favouring the in-group serves people’s self-esteem and may arise by a favourable treatment of the in-group (as noted above), but also by an unfavourable treatment of a marginalized out-group (e.g., substance abusers; underestimating the likelihood of positive outcomes/overestimating the likelihood of negative outcomes).

Importantly, the effects reported here arose by our participants simply considering group membership. In no case did we inform our participants of particular traits, attitudes, preferences, or other attributes of the three different target characters. By this, we intended to activate specific perceptions of warmth and competence as well as particular attitudes and emotions toward ‘the’ prototypical member of the different groups. For the in-group the prototypical member was supposed to be characterized by high perceived warmth and high perceived competence (according to the SCM/BIAS Map). Future examinations should include the introduction of an in-group member characterized by low moral (i.e., low warmth) and low competence to determine whether social group attributes or specific perceptions of warmth and competence in a social target exert the strongest influence on belief formation and updating.

In conclusion, our study revealed that social stereotypes may considerably influence future expectancies for others and their modification. As such, the phenomenon of asymmetrical belief update, which helps generate and maintain personal optimism bias, also extends to social targets.

## Additional File

The additional file for this article can be found as follows:

10.5334/irsp.647.s1 Supplementary Material. Additional information and analyses.

## Footnotes

The original task included a fourth character (a businessperson) that represented a high competence-low warmth out-group member. For time concerns, and because we did not obtain consistent findings across studies for this character (small optimism bias, Dricu, Schüpbach, et al., 2020, versus no bias, Dricu et al., 2018), the businessperson was not included in the present research.

Results do not change if we additionally include the following covariates: perceived warmth, perceived competence, and degree of identification with each character.

## Appendix

**Table A1:** The target events used in the experiment.

HYPOTHETICAL EVENT	VALENCE *M* (*SD*)	CONTROLLABILITY *M* (*SD*)	EMOTIONAL AROUSAL *M* (*SD*)	EXPERIENCE *M* (*SD*)	FEEDBACK/BASE RATE

Look after a child for two hours and the child has fun doing so	79.54 (15.60)	79.12 (14.41)	66.40 (19.57)	64.52 (28.71)	67

Take a hot bath or shower on a cold day	85.74 (13.32)	92.27 (10.37)	50.77 (13.61)	81.83 (17.15)	68

Win a sports bet	73.51 (18.95)	21.68 (19.26)	67.34 (18.81)	8.26 (18.21)	40

Meet an old friend on the street by chance	79.20 (13.46)	18.51 (20.05)	68.40 (18.19)	64.37 (21.05)	69

Successful speech delivery	79.79 (18.06)	82.06 (13.54)	69.43 (19.00)	53.72 (25.38)	47

Friend returns previously borrowed money	72.39 (13.98)	57.93 (18.88)	43.76 (16.37)	65.46 (16.25)	60

Win a karaoke contest	75.13 (15.49)	60.52 (19.59)	63.04 (18.81)	12.66 (21.35)	44

Hear a funny joke	77.66 (14.12)	42.07 (21.24)	57.17 (17.50)	76.88 (17.16)	72

New neighbor comes to introduce themselves	72.13 (14.73)	27.18 (20.93)	52.83 (20.40)	39.05 (25.52)	62

Listen to your favorite song on the radio	77.43 (13.99)	36.80 (27.23)	57.73 (20.34)	74.57 (19.49)	68

Being hugged by a friend	87.73 (10.91)	66.11 (18.73)	79.00 (11.70)	90.89 (13.78)	71

Relatives greet you at a family gathering	82.18 (14.09)	59.87 (19.49)	72.63 (19.17)	83.63 (17.58)	69

Lose 50 CHF on the street	24.06 (23.21)	58.26 (30.06)	63.63 (25.51)	22.24 (23.27)	50

Computer crashes while writing a document	14.73 (12.52)	31.23 (22.51)	68.15 (19.09)	54.78 (22.24)	56

Marriage ends in a bitter divorce	6.06 (12.66)	62.51 (17.92)	96.57 (16.25)	5.89 (18.17)	45

Find rotten food in the fridge	25.55 (14.62)	87.56 (13.75)	36.27 (20.98)	55.29 (16.45)	62

Being heartbroken at the end of a relationship	6.62 (9.82)	34.24 (23.83)	97.48 (19.95)	51.50 (26.22)	68

Shop closes just when you arrive	30.83 (13.48)	79.34 (21.05)	45.93 (19.01)	51.67 (22.51)	60

Drink cold coffee or tea	40.28 (15.47)	84.83 (16.84)	26.32 (18.94)	62.66 (24.52)	60

The neighbor listens to very loud music	33.27 (13.75)	30.20 (20.98)	50.00 (19.72)	47.10 (24.45)	62

Use an unhygienic public toilet	40.01 (14.11)	71.76 (22.86)	25.48 (18.46)	80.16 (18.41)	70

Get a wound that needs stitches	17.35 (14.40)	57.88 (21.50)	62.11 (21.88)	32.72 (29.74)	64

Have a hardly bearable toothache	9.57 (11.35)	45.12 (23.26)	56.74 (19.93)	33.18 (25.16)	71

Being mistaken for another person on the street	46.07 (9.73)	13.06 (18.78)	37.11 (17.80)	48.49 (24.91)	53

*Note*: The twelve positive and twelve negative events had been chosen to be balanced on their values (0–100) of perceived valence (very negative/very positive), controllability (very uncontrollable/very controllable), emotional arousal (not arousing at all/very arousing), personal experience (no experience at all/a lot of experience) and base rate (not frequent at all/very frequent). The values represent mean ratings based on a sample of 89 respondents. Standard deviations are presented in parentheses. The full set can be found in (Dricu et al., 2018).
