# Supplementary material for: Asymmetrical Update of Beliefs About Future Outcomes is Driven by Outcome Valence and Social Group Membership
Source: Int Rev Soc Psychol. 2023 Apr 17;36:2. doi: 10.5334/irsp.647 (PMC12372664; doi:10.5334/irsp.647)
Supplement: Supplementary Material. — Additional information and analyses. [file irsp-36-647-s1.pdf]

Supplementary Material

Asymmetrical Update of Beliefs About Future Outcomes

is Driven by Outcome Valence and Social Group Membership

1 Supplementary Analysis S1: Warmth, competence and identification with the characters

As a manipulation check for whether the assumptions of designated warmth and competence would hold in our sample, we analysed the scores of perceived warmth and competence of each of the three characters. Repeated-measures ANOVA with the factor character (three levels: in-group, mild out-group, extreme out-group) on scores of perceived *warmth* showed a statistically significant difference between characters, $F(2, 264) = 547.29$, Greenhouse-Geisser correction, $p < .001$, $\eta_p^2 = .82$. Post-hoc tests with the Bonferroni correction revealed that all characters differed in perceived competence (all $p < .001$): the mild out-group character ($M = 85.58$) was rated the warmest, followed by the in-group character ($M = 73.37$; p < .001) and the extreme out-group character ($M = 31.70$, p < .001). Repeated-measures ANOVA with the factor character (three levels: in-group, mild out-group, extreme out-group) on scores of perceived *competence* showed a significant difference between characters, $F(2,264) = 517.93$, sphericity assumed, $p < .001$, $\eta_p^2 = .80$. Post-hoc tests with the Bonferroni correction revealed that all characters differed in perceived competence (all $p < .001$): the in-group character ($M = 77.17$) was rated the highest in competence, followed by the mild out-group character ($M = 61.40$) and the extreme out-group character ($M = 20.41$).

To determine whether the participants identified with the student characters as hypothesized and, thus, whether the in-group-out-group manipulation worked, we analyzed the ratings in the IOS task. A one-way repeated-measures ANOVA with the factor character (three levels: in-group, mild out-group, extreme out-group) on scores of the Inclusion of Other in the Self measure yielded significant differences in how much participants identified with each character, $F(2, 264) = 344.42$, $p < .001$, $\eta_p^2 = .72$. Pairwise comparisons using the Bonferroni correction revealed that all characters were rated significantly different from each other (all $p < .001$): the participants identified the most with the in-

group character (*M* = 5.70), followed by the mild out-group character (*M* = 3.31) and the extreme out-group character (*M* = 1.75).

2 Supplementary Figure S1

Histogram of base rates for positive and negative events.

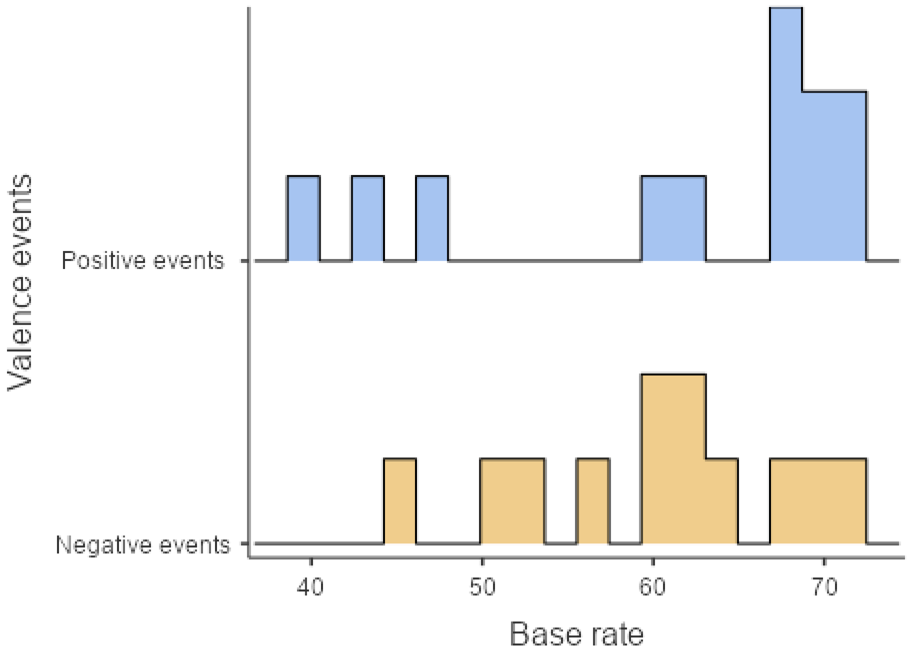

3 Supplementary Table S1

Model information for the Linear Mixed Model.

Method	Linear mixed model fit by REML
Formula	Update ~ 1 + Character + Valence_scenario + Valence_feedback + Estimation_error + Estimate1 + Character:Valence_scenario + Character:Valence_feedback + Valence_scenario:Valence_feedback + Estimation_error:Character + Estimate1:Character + Character:Valence_scenario:Valence_feedback+(1 | Scenario_ID)+(1 | Subject_ID)

4 Supplementary Table S2

Formulas computing the update values.

Positive events, good news = (Estimate 2 – Estimate 1)
Positive events, bad news = (Estimate 1 – Estimate 2)
Negative events, good news = (Estimate 1 – Estimate 2)
Negative events, bad news = (Estimate 2 – Estimate 1)

Note. Computed at the level of each participant and each experimental trial.

5 Supplementary Analysis S2: Determining the likelihood of the null findings for positive events

To determine whether the null effects for the in-group and the mild out-group were credible for the belief update in positive situations, we performed a series of post-hoc Monte Carlo simulations. These simulations determined the minimum effect size necessary to find significance of the feedback main effect (update to good news vs. bad news) for the in-group and mild out-group – despite a character × main-effect interaction. This meant keeping the inverse effect for the extreme out-group that was present in the original dataset (stronger updates for bad than good news).

A custom-built Matlab script took the original data setup but used the random number generator function to replace all data points (see Supplementary **Table S3** for the weights used for the simulations). Estimation error was created randomly using the overall mean and standard deviations of the actual sample. The size of update was also created randomly using the Matlab copularnd function but with the property of being correlated to the estimation error (because this correlation was also present in the actual data set). The initial estimate was the difference of estimation error and feedback, which had been retained from the original data. Like in the actual experiment, the "good news" and the "bad news" labels as well as the estimation errors were consequences of the original likelihood estimations (simulated here using random values) and the given values of feedback (copied here from the actual experiment). We then performed simulations with increasing effect sizes as measured in standardized mean differences (standard deviations) for the magnitude of the update (results in Supplementary **Table S4**).

The simulations with zero change/zero effect size can be considered a sanity check **(**Supplementary **Table S4**). This sanity check shows that all effects of interest were between 3% and 6% (except for the estimation error, which was significant by design), meaning that the method is not likely

to overestimate the importance of null effects, while still capable of detecting small and medium effects for main effects and two-way interactions.

Supplementary Table S3

Effect sizes in standard deviations used for the Monte Carlo simulations for the target characters in-group, mild out-group, and extreme out-group, respectively. These effect sizes follow the pattern of results from the actual dataset.

**Main Effect Feedback**	**Main Effect Scenario**	**Character x Feedback interaction**	**Positive Scenario**	**Positive Scenario**	**Negative Scenario**	**Negative Scenario**
**(by Character)**			**Good News**	**Bad News**	**Good News**	**Bad News**
0/0/0	0/0/0	0	0/0/0	0/0/0	0/0/0	0/0/0
0.05/0.05/-0.05	0/0/0	0.1	0.05/0.05/0	0 /0/0.05	0.05/0.05/0	0 /0/0.05
0.10/0.10/-0.10	0/0/0	0.2	0.10/0.10/0	0 /0/0.10	0.10/0.10/0	0 /0/0.10
0.15/0.15/-0.15	0/0/0	0.3	0.15/0.15/0	0 /0/0.15	0.15/0.15/0	0 /0/0.15
0.20/0.20/-0.20	0/0/0	0.4	0.20/0.20/0	0 /0/0.20	0.20/0.20/0	0 /0/0.20
0.25/0.25/-0.25	0/0/0	0.5	0.25/0.25/0	0 /0/0.25	0.25/0.25/0	0 /0/0.25
0.30/0.30/-0.30	0/0/0	0.6	0.30/0.30/0	0 /0/0.30	0.30/0.30/0	0 /0/0.30
0.35/0.35/-0.35	0/0/0	0.7	0.35/0.35/0	0 /0/0.35	0.35/0.35/0	0 /0/0.35
0.40/0.40/-0.40	0/0/0	0.8	0.40/0.40/0	0 /0/0.40	0.40/0.40/0	0 /0/0.40
0.45/0.45/-0.45	0/0/0	0.9	0.45/0.45/0	0 /0/0.45	0.45/0.45/0	0 /0/0.45
0.50/0.50/-0.50	0/0/0	1	0.50/0.50/0	0 /0/0.50	0.50/0.50/0	0 /0/0.50

Note. Positive main effects of feedback refer to a greater update for good news vs. bad news. Negative main effects of feedback refer to a greater update for bad news than good news.

Supplementary Table S4

Progressive effect sizes of update for good news vs. bad news in positive situations for the in-group and mild out-group.

**Condition**	**0**	**0.025**	**0.05**	**0.075**	**0.1**	**0.125**	**0.15**	**0.175**	**0.2**	**0.225**	**0.25**
Character	5%	5%	8%	14%	26%	37%	51%	65%	77%	88%	94%
Scenario	4%	5%	4%	4%	5%	4%	4%	5%	4%	5%	5%
Feedback	3%	6%	10%	20%	35%	51%	65%	80%	88%	95%	98%
Initial Estimate	5%	5%	5%	4%	5%	5%	5%	5%	5%	4%	5%
Estimation error	100%	100%	100%	100%	100%	100%	100%	100%	100%	100%	100%
*Character*Scenario*	5%	5%	7%	6%	6%	6%	6%	6%	6%	6%	7%
*Character*Feedback*	4%	10%	17%	40%	65%	83%	95%	99%	100%	100%	100%
Scenario Feedback*	3%	4%	3%	4%	4%	4%	4%	5%	5%	4%	5%
Character Initial Estimate*	4%	5%	5%	5%	5%	4%	5%	4%	4%	5%	7%
Character Estimation error*	3%	4%	5%	4%	4%	4%	4%	4%	5%	5%	5%
*Character *Scenario *Feedback*	5%	5%	6%	6%	7%	6%	5%	7%	6%	5%	7%

Note. Upper row displays progressive effect sizes (standardized mean differences in update for good news vs. bad news). Percentages in each column refer to the percentage of 1000 Monte Carlo simulations that were significant at $p < .05$ to detect the corresponding progressive effect sizes.

6 Main analyses: Hypotheses-unrelated effects

In this section, we report effects that are not directly related to our hypotheses: Our LMM yielded no main effect of valence of scenario, $F(1,23.3) = .09$, $p = .764$ (**Table 3**), but there was a main effect of valence of feedback, $F(1,8434.3) = 69.03$, $p < .001$ (**Table 3**), meaning that participants were significantly more willing to update in response to good news ($M = 8.43\%$, $SE = .42$) than bad news ($M = 5.79\%$, $SE = .41$). Importantly though, this main effect of valence of feedback was qualified both by valence of scenario and by character, as described above for the significant three-way interaction, demonstrating that it did not hold across characters and event valence levels.

There further was a main effect of estimation error, $F(1,9288.7) = 763.74$, $p < .001$ (**Table 3**), meaning that respondents generally updated increasingly more as their initial estimates increasingly departed from the provided feedback/base rate. The interactions between character and estimation error, $F(2, 9098.3) = .95$, $p = .385$, and between character and the initial estimate, $F(2,9353.4) = .76$, $p = .470$, were not significant. The lack of a significant interaction between the character and the estimation error suggests that, although the character influences the initial estimates (see Supplementary **Analysis S3** and Supplementary **Figure S2**), the effect of the estimation errors on updates did not differ among the three different characters. Interestingly as well, the differential first estimates (i.e., influenced by the character) did not influence the amount of update, $F(1,1072.77) = 1.07$, $p = .301$.

The remaining two-way interactions were altogether statistically significant but qualified by the significant three-way interaction described before: character × feedback, $F(2,8707.5) = 15.65$, $p < .001$, character × scenario, $F(2,9387.0) = 6.55$, $p = .001$, and feedback × scenario, $F(1,3678.5) = 66.05$, $p < .001$ (**Table 3**). In the name of replicability, we also provide the results

for the desirability (i.e., optimism) bias before and after the feedback (Supplementary **Analysis S4** and Supplementary **Figure S3**), in line with the methods described in (Dricu et al., 2018; Dricu, Schüpbach, et al., 2020).

7 Supplementary Analysis S3: Initial estimates

A linear mixed model was conducted on the first estimates as the dependent variable and the following predictors: character (in-group, mild out-group, extreme out-group), valence of scenario (positive and negative) and the interaction term character × valence of scenario. There was a main effect of character, $F(2,9415.05) = 258.94$, $p < .001$, qualified by an interaction between character and valence of scenario, $F(2,9415.05) = 518.31$, $p < .001$; **Figure S2**. The post-hoc tests revealed significantly different likelihood estimates between all characters for both positive and negative scenarios (all $p < .029$, Bonferroni correction for multiple testing). For positive events, respondents rated the in-group with the highest likelihood (M =71.95%), followed by the mild out-group (M = 64.1%) and the extreme out-group (M = 44.86%). For negative events, respondents rated the extreme out-group with the highest likelihood (M = 63.97%), followed by the in-group (M = 61.44%) and the mild out-group (M = 49.04%). The main effect of valence of scenarios was not significant, $F(2,4579.86) = 3.35$, p =.067.

Figure S2

Differences in the first estimates between characters, separately for positive and negative scenarios.

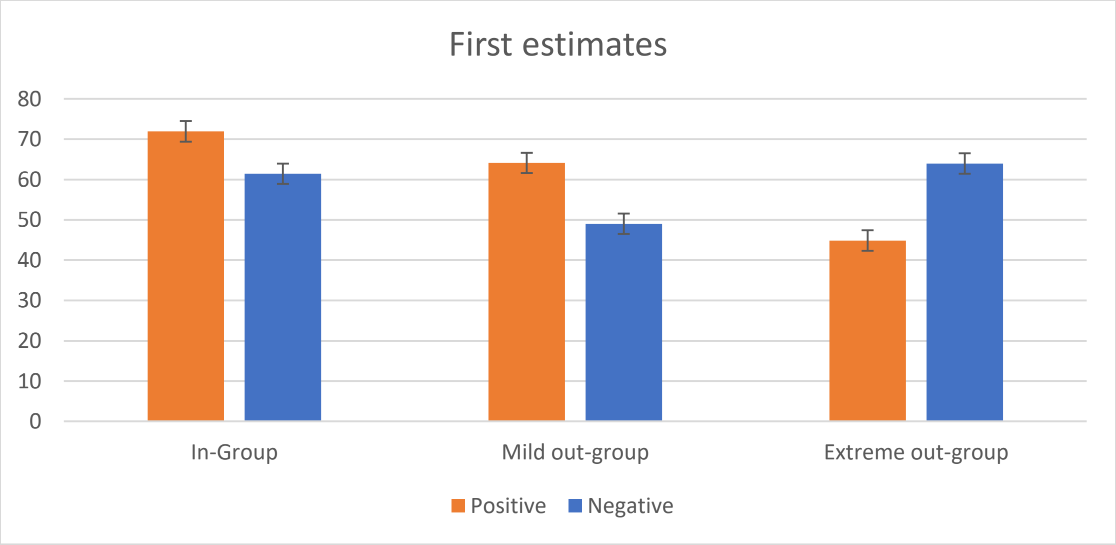

Note. All characters were rated significantly different within positive and negative scenarios (p <.029, Bonferroni correction for multiple testing).

8 Supplementary Analysis S4: Desirability bias (likelihood positive events – likelihood negative events)

A 3 (character) x 2 (feedback: before and after) repeated-measures ANOVA on the desirability bias scores revealed a main effect of character, $F(2,208) = 232.410$, $p < .0005$, $\eta_p^2 = .691$, a main effect of feedback, $F(1,104) = 20.011$, $p < .0005$, $\eta_p^2 = .161$, and an interaction between character and time, $F(2,208) = 23.742$, $p < .0005$, $\eta_p^2 = .186$ (Supplementary **Figure S3)**. One sample t-tests showed that respondents expected significantly more positive outcomes than negative outcomes for the in-group, $t(104) = 14.051$, $p < .0005$, and the mild out-group, $t(104) = 17.068$, $p < .0005$, but the opposite for the extreme out-group, $t(104) = -8.822$, $p < .0005$, in line with previous results (Dricu et al., 2018; Dricu et al., 2020). Interestingly, the magnitude and the direction of the bias was unaffected by the delivery of feedback values for the in-group, $t(104) = .134$, $p = .893$, and the mild out-group, $t(104) = .339$, $p = .736$. However, the size of the desirability bias for the extreme out-group was lowered by the delivery of the feedback, $t(104) = -7.149$, $p < .0005$ (desirability bias before feedback: $M = -14.381$, $SD = 16.704$, desirability bias after feedback: $M = -8.305$, $SD = 16.037$).

Figure S3

The size of the desirability bias (likelihood positive events – likelihood negative events) before and after receiving the feedback.

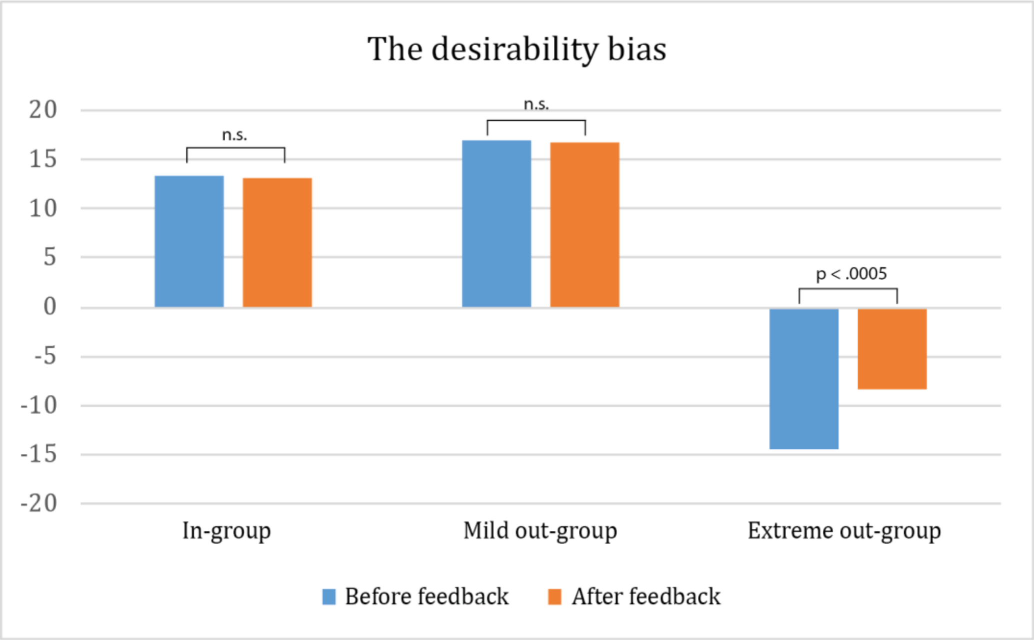

References

Dricu, M., Bührer, S., Hesse, F., Eder, C., Posada, A., & Aue, T. (2018). Warmth and competence predict overoptimistic beliefs for out-group but not in-group members. *PloS One*, *13*(11), e0207670.

Dricu, M., Schüpbach, L., Bristle, M., Wiest, R., Moser, D. A., & Aue, T. (2020). Group membership dictates the neural correlates of social optimism biases. *Scientific Reports*, *10*(1), 1-17.
